# Serotype-Independent Protection Against Invasive Pneumococcal Infections Conferred by Live Vaccine With *lgt* Deletion

**DOI:** 10.3389/fimmu.2019.01212

**Published:** 2019-05-29

**Authors:** A-Yeung Jang, Ki Bum Ahn, Yong Zhi, Hyun-Jung Ji, Jing Zhang, Seung Hyun Han, Huichen Guo, Sangyong Lim, Joon Yong Song, Jae Hyang Lim, Ho Seong Seo

**Affiliations:** ^1^Research Division for Biotechnology, Korea Atomic Energy Research Institute, Jeongeup, South Korea; ^2^Department of Internal Medicine, Korea University College of Medicine, Seoul, South Korea; ^3^Department of Radiation Science and Technology, University of Science and Technology, Daejeon, South Korea; ^4^DRI and BK21 Plus Program, Department of Oral Microbiology and Immunology, School of Dentistry, Seoul National University, Seoul, South Korea; ^5^State Key Laboratory of Veterinary Etiological Biology, National Foot and Mouth Disease Reference Laboratory, Lanzhou Veterinary Research Institute, Chinese Academy of Agricultural Sciences, Lanzhou, China; ^6^Department of Microbiology, Ewha Womans University College of Medicine, Seoul, South Korea

**Keywords:** *Streptococcus pneumoniae*, Lgt, lipoprotein, live attenuated vaccine, mucosal immunity

## Abstract

*Streptococcus pneumoniae* is the most common respiratory bacterial pathogen among cases of community-acquired infection in young children, older adults, and individuals with underlying medical conditions. Although capsular polysaccharide-based pneumococcal vaccines have contributed to significant decrease in invasive pneumococcal infections, these vaccines have some limitations, including limited serotype coverage, lack of effective mucosal antibody responses, and high costs. In this study, we investigated the safety and immunogenicity of a live, whole-cell pneumococcal vaccine constructed by deleting the gene for prolipoprotein diacylglyceryl transferase (*lgt*) from the encapsulated pneumococcal strain TIGR4 (TIGR4Δ*lgt*) for protection against heterologous pneumococcal strains. Pneumococcal strain TIGR4 was successfully attenuated by deletion of *lgt*, resulting in the loss of inflammatory activity and virulence. TIGR4Δ*lgt* colonized the nasopharynx long enough to induce strong mucosal IgA and IgG2b-dominant systemic antibody responses that were cross-reactive to heterologous pneumococcal serotypes. Finally, intranasal immunization with TIGR4Δ*lgt* provided serotype-independent protection against pneumococcal challenge in mice. Taken together, our results suggest that TIGR4Δ*lgt* is an avirulent and attractive broad-spectrum pneumococcal vaccine candidate. More broadly, we assert that modulation of such “master” metabolic genes represents an emerging strategy for developing more effective vaccines against numerous infectious agents.

## Introduction

*Streptococcus pneumoniae* (*Sp*, also known as pneumococcus) is the most common bacterial pathogen causing human diseases such as otitis media, pneumonia, and life-threatening invasive pneumococcal diseases, including meningitis and sepsis. It is responsible for an estimated 900,000 cases of pneumonia, 40,000 cases of invasive pneumococcal disease, and 4,000 associated deaths annually in the U.S. alone (1). *Sp* asymptomatically colonizes the nasopharynx of ~60% of the population and disseminates to cause invasive disease under immunocompromised conditions and co-infection with respiratory viral infections (2). Thus, immunization against pneumococcus is strongly recommended for populations at risk, including young children, the elderly, and patients with underlying medical conditions (3). A mucosal protective immune response against pneumococcus may be the most effective strategy for preventing opportunistic and invasive infections by eliminating early pneumococcal colonization of the nasopharynx (4, 5).

Currently, two types of licensed pneumococcal vaccines are available, both of which are based on the generation of antibodies against pneumococcal capsular polysaccharides (6). A 23-valent pneumococcal PS vaccine (PPV23), composed of 23 different capsular polysaccharide from commonly encountered serotypes, is widely recommended for older adults but not for young children due to its low immunogenicity in infants (7). A protein-conjugated capsular polysaccharide vaccine (PCV) was initially developed to provide protection against the seven most prevalent serotypes causing invasive pneumococcal disease in young children. This was later expanded to include 10 or 13 of the most invasive pneumococcal serotypes and is highly immunogenic in both adults and infants (8). A dramatic decrease in the burden of invasive pneumococcal diseases has been reported in many countries in which PCVs are in widespread use (9, 10). However, both vaccines are complex and costly to manufacture, making them inaccessible for low-income populations. Additionally, these serotype-based vaccines have significant limitations in serotype coverage, resulting in serotypes being increasingly replaced by non-vaccine serotypes or the emergence of new serotypes and non-typeable pneumococcal strains that cause invasive pneumococcal disease (11–13). Although the overall incidence of invasive pneumococcal disease has declined by 47% as a result of the introduction of PCVs, the prevalence of non-vaccine serotypes (21, 23B, 33F, and 35F) has significantly increased among asymptomatic carriers and invasive pneumococcal diseases in countries in which a PCV is used nationwide (3, 14). In addition, both PPVs and PCVs have recently been reported to produce diminished mucosal immune responses, especially in the production of immunoglobulin A (IgA), a predominant Ig isotype at the respiratory surface commonly colonized by pneumococcus (15). Due to these limitations, inactivated and live whole-cell-based vaccines have been extensively studied as cost-effective and broad-spectrum vaccine candidates at the clinical and pre-clinical levels (16–19). Also, a low cost of production and an ease of storage and handling of whole cell vaccines compared to sophisticated alternative vaccines, such as PCV and PPV, would facilitate the widespread use of them in the developing and undeveloped countries, where the vaccination for pneumococcus is greatly limited. Likewise, the development of inactivated whole-cell-based vaccines for pneumococcus has been ongoing in both clinical and preclinical trials since 1911, and recent meta-analyses have affirmed their efficacy (18, 20).

As another approach for developing whole-cell-based vaccines, live attenuated vaccines are advantageous, as they enhance humoral and mucosal immune responses following a single-dose vaccination, in contrast to inactivated whole-cell-based vaccines (4, 21). However, the main obstacle to the development of live attenuated vaccines is achieving satisfactory attenuation without compromising immunogenicity. To date, most live attenuated pneumococcal vaccine strains have been constructed in a capsule-negative background and carry additional mutations in important virulence genes, such as *pspA, ply*, and *lytA* (21, 22). However, these live attenuated vaccines may be suboptimally immunogenic due to the deletion of several virulence genes that also induce protective immune responses. To overcome such drawbacks, central regulatory or component “master” genes have been used as alternative targets for live attenuated vaccine development, since their deletion may downregulate, but not necessarily silence the expression of many virulence and metabolic genes (19, 23). For example, a pneumococcal mutant strain deficient in the gene *ftsY*, which encodes a central component of the signal recognition particle (SRP) pathway responsible for delivering membrane and secretory proteins, was highly protective against multiple serotypes in mouse models of pneumococcal otitis media and invasive pneumococcal disease by bolstering IgG2 serum responses (21).

Lipoprotein diacylglyceryl transferase (Lgt) is a highly conserved, commitment-step enzyme that catalyzes diacylglyceryl attachment to an N-terminal cysteine residue in a consensus peptide sequence known as a pre-prolipoprotein on the membrane (24, 25). It is essential for growth in most Gram-negative bacteria but not in *Streptococcus* spp. or *Staphylococcus* spp. (26). Lipoproteins are membrane-anchoring proteins, which locates beneath peptidoglycan layer in Gram-positive bacteria, with a variety of physiological and pathogenic functions in bacteria, including roles in cell division, conjugation, nutrient acquisition, adhesion, invasion, and immune evasion. In addition, the lipoprotein lipid moiety significantly contributes to the activation of Toll-like receptor 2 (TLR2)-mediated host innate immune responses (27–29). Following the diacylation of pre-prolipoproteins by Lgt, the N-terminal signal peptide is cleaved off by lipoprotein signal peptidase II (Lsp), resulting in a mature lipoprotein (25, 30, 31). Deletion of *lgt* in Gram-positive bacteria can strongly attenuate virulence, resulting in improper lipoprotein anchoring in the cytoplasmic membrane (32–36). A previous study also showed that a pneumococcal mutant strain deficient in *lgt* exhibited reduced TLR2-mediated inflammatory responses *in vitro* and diminished invasiveness *in vivo* (37). Since *lgt*-mutant pneumococcal strains exhibit greatly diminished virulence while still expressing many surface antigens, we hypothesized that a novel intranasal vaccine based on an *lgt*-mutant strain would be a promising and effective pneumococcal live attenuated vaccines candidate.

In this study, we aimed to develop an encapsulated pneumococcal TIGR4 strain deficient in *lgt* (TIGR4Δ*lgt*) and evaluate its efficacy in protecting against infection from heterologous pneumococcal strains in mice.

## Materials and Methods

### Reagents

All antibodies, purified proteins, antibiotics, and other reagents used in this study were purchased from Sigma-Aldrich (St. Louis, MO, USA). The mouse TNF-α enzyme-linked immunosorbent assay (ELISA) kit was purchased from eBioscience (San Diego, CA, USA). An anti-PsaA monoclonal antibody was kindly provided by Prof. Nahm (University of Alabama at Birmingham, AL, USA).

### Bacterial Strains and Generation of Supernatants and Crude Extracts

The bacteria and plasmids used in this study are listed in Supplement Table 1. *Streptococcus pneumoniae* (*Sp*) strain TIGR4 was obtained from the American Type Culture Collection (ATCC; Manassas, VA, USA). *Sp* serotypes 2 (D39), 3 (wu2), 6B, 9V, 19F, and 23F were kindly provided by Prof. Nahm (University of Alabama at Birmingham). Pneumococcal strains were grown in Todd-Hewitt broth (THB; Difco, Franklin Lakes, NJ, USA) supplemented with 0.5% yeast extract (Difco). At mid-log phase [optical density at 600 nm (OD_600_) of 0.3–0.4], bacteria were pelleted by centrifugation, and culture supernatants were filtered through a 0.22-μm membrane filter (Merck Millipore, Billerica, MA, USA). Bacterial pellets were resuspended in phosphate-buffered saline (PBS; Lonza, Basel, Switzerland).

### Western Blotting Analysis

Bacterial culture supernatants were loaded and separated on Bis-Tris Bolt sodium dodecyl sulfate polyacrylamide gels (Invitrogen, Carlsbad, CA, USA), followed by transfer to nitrocellulose membranes (Bio-Rad, Hercules, CA, USA). The membranes were blocked with 5% dry skim milk (Bio-Rad) in PBS/0.05% Tween-20 (PBS-T) and then incubated with anti-PsaA monoclonal antibody (Xir-126) or anti-phosphocholine monoclonal antibody (TEPC-15, Sigma-Aldrich). The membrane was then washed with PBS-T and incubated with a horseradish peroxidase-conjugated rabbit anti-mouse immunoglobulin (Southern Biotech, Birmingham, AL, USA). Membrane-bound peroxidase was detected with 3,3′,5,5′-tetramethylbenzidine substrate solution (Thermo-Fisher Scientific, Waltham, MA, USA).

### Construction of *lgt*-Deficient TIGR Strain

Primers used in this study are listed in Supplement Table 2. A gene replacement cassette was constructed by cloning the chromosomal regions flanking *lgt* and inserting them upstream and downstream of the *kan* gene in pK326, as described previously (38). A 1,023-bp upstream segment and a 930-bp downstream segment were amplified using paired primers (LGT-UpF/LGT-UpR and LGT-DnF/LGT-DnR, respectively). Amplified fragments were then cloned sequentially into the pK326 plasmid, resulting in the pKO-lgt plasmid, which was then introduced into TIGR4 by natural transformation, as previously described (39). In brief, a culture of TIGR4 was diluted 100-fold in fresh transformation THB supplemented with 2% bovine serum albumin (BSA; Sigma-Aldrich), 0.1% CaCl_2_ (Sigma-Aldrich), and 200 ng/ml competence-stimulating peptide. After incubation at 37°C for 14 min, cells were mixed with ~5 μg of pKO-lgt, followed by an additional 2 h of incubation at 37°C. Transformed cells were then plated onto blood agar plates containing 300 μg/ml kanamycin (Sigma-Aldrich) for selection of the TIGR4Δ*lgt* strain.

### Inactivation of *lgt*-Deficient TIGR Strain

Harvested TIGR4Δ*lgt* (1 × 10^10^ CFU/ml) were irradiated using a ^60^Co-gamma irradiator (point source AECL, IR-79, MDS Nordion International Co., Ottawa, ON, Canada) at the Advanced Radiation Technology Institute of Korea Atomic Energy Research Institute (Jeoneup, Korea) with absorbed dose of 5 kGy for 1 h at room temperature.

### Mammalian Cells and Culture Conditions

Mouse RAW264.7 cells (TIB-71) were obtained from ATCC. RAW264.7 cells were cultured in Dulbecco's modified Eagle's medium (Cellgro Mediatech, Herndon, VA, USA) supplemented with 10% heat-inactivated fetal bovine serum (HyClone, Logan, UT, USA), 100 units/ml penicillin, and 100 μg/ml streptomycin at 37°C in a humidified incubator with 5% CO_2_.

### Measurement of Nitrite and TNF-α

Thioglycollate-elicited peritoneal cells were collected from wild-type (WT), ΔTLR2, and ΔTLR4 male C57BL/6 mice at 6 weeks of age provided kindly from Prof. Suzanne Michalek (University of Alabama at Birminghum). Mouse primary peritoneal cells or RAW264.7 cells were dispensed into 96-well plates (SPL, Suwon, Korea) at a density of 2 × 10^5^ cells/ml and stimulated with TIGR4 or TIGR4Δ*lgt* for 12 h. For nitrite measurement, 100 μl of culture supernatant was mixed with an equal volume of Griess reagent (1% sulfanilamide, 0.1% naphthylethylenediamine dihydrochloride, and 2% phosphoric acid) and incubated at RT for 5 min. The optical density was then measured at a wavelength of 540 nm using a Victor X3 light plate reader (Perkin-Elmer, Waltham, MA, USA). NaNO_2_ solution was used to generate the standard curve. The amount of TNF-α in the cell culture supernatant was determined with a commercially available sandwich-type ELISA (eBioscience) according to the manufacturer's protocol.

### Purification of Lipoteichoic Acid (LTA)

Pneumococcal LTA was prepared using organic solvent extraction, octyl-Sepharose, and ion-exchange chromatography, as previously described (40). Levels of endotoxin contamination were determined using the QCL-1000 quantitative chromogenic Limulus Amoebocyte Lysate (LAL) assay (Bio-Whittaker, Walkersville, MD, USA) according to the manufacturer's instructions.

### Microarray Analysis

To examine the effect of the loss of lipoprotein biosynthesis on global gene expression in *Sp*, microarray analysis was performed to compare transcript levels between WT TIGR4 and TIGR4Δ*lgt*, as described previously (41). Total bacterial RNA was isolated from cells grown statically to mid-log phase (OD_600_ = ~0.35) using an SV total RNA isolation kit (Promega, Madison, WI, USA) according to the manufacturer's instructions. A total of 75 μg RNA with 3 μg random hexamer dissolved in 29.5 μl nuclease free-water was denatured at 65°C for 10 min and then placed on ice. After adding 6 μl of 0.1 M dithiothreitol, 12 μl first-strand buffer, 1.5 μl dNTP mix (25 mM dATP, 25 mM dGTP, 25 mM dCTP, 10 mM dTTP), 4 μl Superscript II^®^ reverse transcriptase (Invitrogen), 2 μl RNasin (Promega), and 4 μl of either Cy3- or Cy5-conjugated dTTP (Amersham Biosciences, Buckinghamshire, UK), the labeling mixture (60 μl) was incubated at 42°C for 2 h and supplemented with 2 μl Superscript II at the end of the first hour. Each probe was then denatured with 10 μl of 1 M NaOH and neutralized with 10 μl of 1 M HCl, followed by purification with a PCR purification kit (Qiagen, Venlo, Netherlands). It was then concentrated with a speed vacuum drier. Each probe separately labeled with Cy3 or Cy5 was resuspended in 20 μl distilled water. Whole-genome TIGR4 oligonucleotide microarray chips were obtained from the J. Craig Venter Institute (Rockville, MD, USA). The array consisted of 70-mer oligonucleotide probes, representing 2060 TIGR4 open-reading frames (ORFs) and 457 ORFs from strains G54 and R6. Each oligonucleotide probe sequence was spotted at least five times on the array. All microarray experiments were performed by eBiogen (Seoul, Korea). In brief, equal volumes of labeled probes (20 μl each) from the TIGR4 and TIGR4Δ*lgt* strains were mixed with 40 μl of 2 × hybridization solution consisting of 50% formamide, 10 × saline-sodium citrate, and 0.2% SDS and denatured by boiling for 5 min. Probes were simultaneously hybridized to a chip overnight at 42°C in a hybridization chamber (Corning, Corning, NY, USA) submerged in water. Scans were performed with a Scan Array 5,000 laser scanner using ScanArray 2.1 software (Packard BioChip Technologies, Billerica, MA, USA). Signal intensity was quantified using QuantArray 3.0 software (Packard BioChip Technologies). The statistical significance of differential expression relative to that in the control was assessed using a paired *t*-test and Bayesian analysis, as Bayesian estimates of within-treatment variation tend to reduce the rate of false positives (42). The threshold for a significant change in gene expression was set at *P* < 0.001. Of the genes passing this threshold, only those with a Bayesian confidence value (B-value) above zero were regarded as being significantly affected by Δ*lgt* mutation. B-values were computed using Cyber-T (cybert.microarray.ics.uci.edu).

### Quantitative Real-Time PCR (qRT-PCR) Analysis

Total RNAs were isolated from log-phase cultures of WT TIGR4 and TIGR4Δ*lgt* using an RNeasy^®^ mini kit (Qiagen) according to the manufacturer's instructions. For real-time PCR analysis, cDNA was synthesized from 1 μg total purified RNA using a Primescript 1st strand cDNA synthesis Kit (Takara Bio Inc., Kyoto, Japan) following the manufacturer's instructions. Primers for various genes were designed using Primer Express v2.0 software and are listed in Supplement Table 2. qRT-PCR amplification was performed with SYBR Premix Ex Taq (Takara Bio Inc.) on a Bio-Rad CFX Real-Time System. PCR reactions were performed with one cycle of 95°C for 5 min; 40 cycles of 95°C for 15 s, 60°C for 30 s, and 72°C for 30 s; and one cycle of 72°C for 10 min. Relative quantification of gene expression was determined by the comparative threshold (ΔΔCt) method. The 16S rRNA gene (*rrsH*) was used as a control to normalize the expression levels of target genes.

### Animal Experiments

All animal experiments conducted in this study were approved by the Committee on the Use and Care of Animals at the Korea Atomic Energy Research Institute (KAERI) and performed according to accepted veterinary standards. Six-week-old male C57BL/6 mice were purchased from Orient Bio Inc. and were intranasally (i.n.) vaccinated twice at 14-day intervals with live or killed TIGR4Δ*lgt*. At 10 days after the second vaccination, blood was collected, and antibodies specific to pneumococcus were measured by ELISA. To examine the protective effect of TIGR4Δ*lgt* vaccination, mice were i.n. infected with pneumococcal strains TIGR4, wu2, or D39 at 10 days after the second vaccination. Mouse survival was monitored for 10 days after infection. Blood, nasal washes, and lungs were collected at the time of death or at the end of the experiment for bacterial counting. Tissues were homogenized in 1.5 ml PBS and passed through a 40-μm mesh strainer, and bacterial numbers were then counted.

### Measurement of Mouse Immunoglobulin Levels

Mouse blood samples were taken every 2 weeks. Serum was isolated via centrifugation and stored at −80°C until use. Pneumococcal strains were cultured in THY (THB with 2% yeast extract) and harvested at mid-log phase. The absorbance of the pneumococcal pellet was adjusted to an OD_600_ of 0.1 by dilution with PBS. Then, 96-well immunoplates (SPL) were coated with 100 μl pneumococcal suspension and incubated overnight at 4°C to allow adherence of bacterial cells. The plates were then washed five times with PBS-T, followed by blocking with 1% BSA in PBS for 1 h at RT. After blocking, diluted serum was added to each well and incubated at RT for 1 h, and unbound antibodies were removed by washing with PBS-T. Appropriate dilutions of goat anti-mouse Ig-HRP (Sigma-Aldrich), goat anti-mouse IgG-HRP (Southern-Biotech), or goat anti-mouse IgM-HRP (Southern-Biotech) were added to wells and incubated for 30 min at RT. After washing the plates five times with PBS-T, 100 μl TMB substrate reagent (BD Biosciences, Franklin Lakes, NJ, USA) was added. When colors developed, 50 μl of 2 N H_2_SO_4_ was added, and the absorbance was measured at 450 nm using a Victor X3 light plate reader (Perkin-Elmer).

### Hematoxylin and Eosin (H&E) Staining

Following i.n. inoculation of WT TIGR4 or TIGR4Δ*lgt*, mouse lungs were harvested at 24 h after inoculation. The left lung of each mouse was fixed with 4% paraformaldehyde prior to paraffin embedding to preserve the pulmonary architecture. Paraffin-embedded tissues were then cut into 4-μm-thick sections and stained with H&E to observe histopathological changes. Tissue images were captured with a light microscope (Eclipse Ci-L; Nikon Corp., Tokyo, Japan) and processed using i-Solution Lite software (iMTechnology, Vancouver, BC, Canada).

### Data Analysis

Data are expressed as mean ± standard deviation (SD). Data in bar graphs and bacterial numbers between groups were compared by unpaired *t*-tests. Mouse survival was analyzed by Kaplan–Meier analysis. The significance of differences was assessed by log-rank test using GraphPad Prism (GraphPad Software, La Jolla, CA, USA). Differences with *P-*values < 0.05 were considered statistically significant.

## Results

### Biological Characteristics of *lgt*-Deficient Pneumococcus

**Figure 1:**
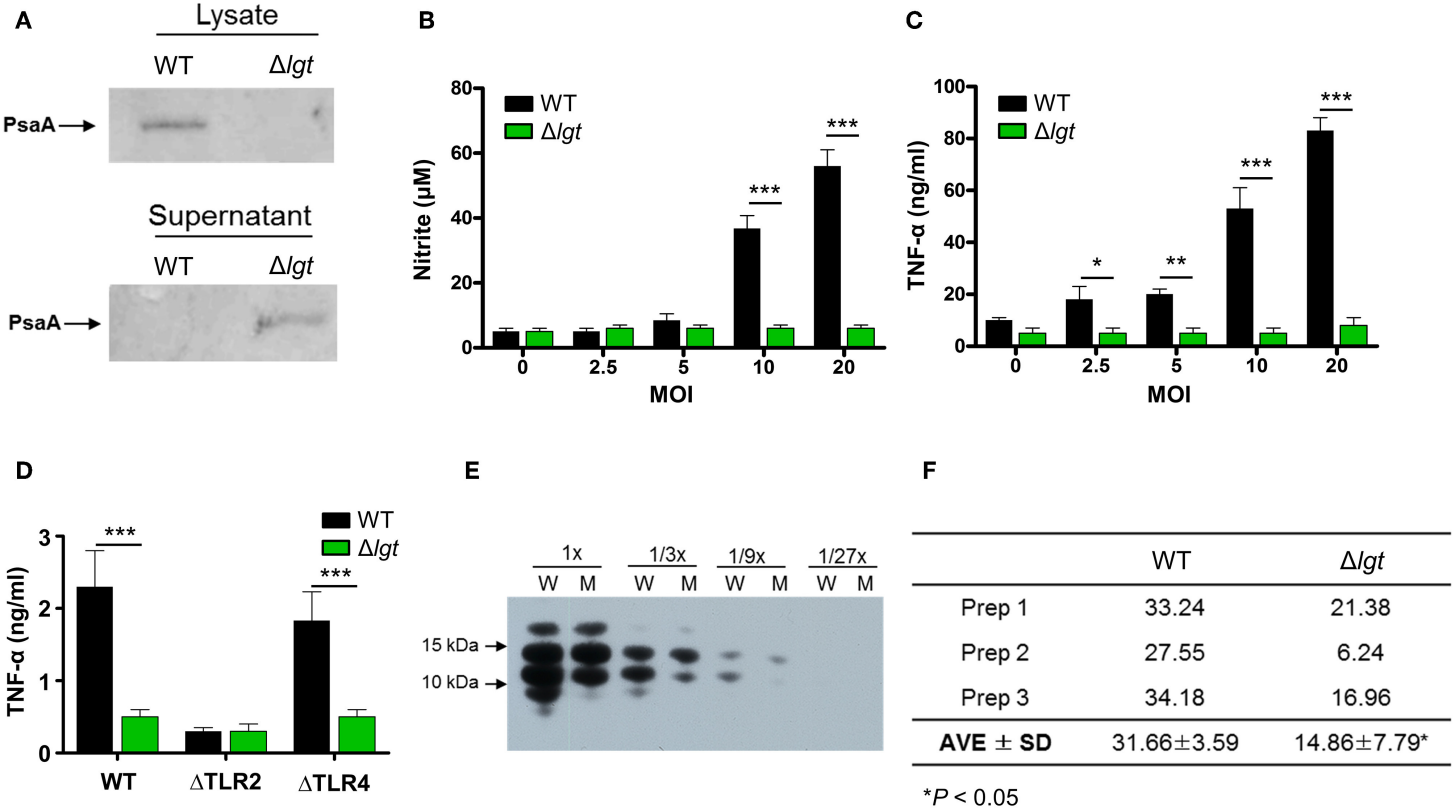
Biochemical and Immunological Characteristics of TIGR4Δ*lgt*. **(A)** Expression of PsaA protein in cell lysates and culture supernatants from wild-type (WT) and *lgt*-deficient (Δ*lgt*) TIGR4 strains. **(B,C)** Nitric oxide **(B)** and TNF-α **(C)** production in culture supernatants of RAW264.7 cells treated with WT or Δ*lgt* strain for 12 h. **(D)** TNF-α production in the culture supernatants of peritoneal cells from wild-type (WT), TLR2-deficient (ΔTLR2), and TLR4-deficient (ΔTLR4) mice infected with WT or Δ*lgt* strain for 12 h. Data in **(B,C)**, and **(D)** represent mean values ± S.D. of triplicate results. ^*^P < 0.05, ^**^P < 0.01, and ^***^P < 0.001 compared to the WT treatment group. **(E)** LTA expression levels in 3-fold serial dilutions of culture supernatants from WT (W) or Δ*lgt* (M) strains. **(F)** Quantification of LTA isolated from the pellets of WT and Δ*lgt* strains. ^*^P < 0.05 compared to the WT group.

To investigate its potential clinical use as a pneumococcal live attenuated vaccine, an *lgt*-deficient pneumococcal strain was constructed from the pneumococcal strain TIGR4 (TIGR4Δ*lgt*). Its biological properties were then examined. First, to verify whether lipoprotein biosynthesis was abrogated in TIGR4Δ*lgt*, the localization of PsaA, a major lipoprotein, was examined in cell lysates and culture supernatants (Figure 1A). As expected, the expression of PsaA was detected in the cell lysate of wild-type (WT) TIGR4, while PsaA accumulated in the culture supernatant of TIGR4Δ*lgt*, indicating its improper anchoring in the membrane. Of note, TIGR4Δ*lgt* grew well in nutrient-rich medium, similar to its parent strain (data not shown).

Since pneumococcus can produce over 32 families of lipoproteins and more than 88 lipoprotein-related proteins (43), it was possible that the *lgt*-deficient mutant would exhibit impairment of cellular functions associated with transporting and synthesizing membrane components. To determine whether the pneumococcal transcriptome was altered by *lgt* deletion, gene expression microarray analysis was performed. In TIGR4Δ*lgt*, 164 genes were upregulated, and 161 genes were downregulated compared to their expression levels in the TIGR4 parent strain (*P* < 0.01). Among these, the mRNA expression levels of previously identified vaccine targets, such as choline binding proteins, lipoproteins, and LPxTG motif cell wall-anchoring proteins were further analyzed (Tables 1, 2). This revealed that, of 18 selected vaccine targets, expression levels of *potD, vgcL, clpP*, and *htrK* were downregulated but not significantly so in TIGR4Δ*lgt*, while the expression of the other genes was similar to or significantly higher than that in TIGR4.

**Table 1:** mRNA expression of candidate vaccine genes in wild-type TIGR4 and TIGR4Δ*lgt* pneumococci.

**Locus tag**	**Gene**	**Ratio (Δ*lgt*/WT)**	***P*-value**	**Description**	**qPCR**	**References**
SP1386	*potD*	0.675	0.074	Spermidine/putrescine ABC transporter	ND	(44)
SP0189		0.717	0.090	Conserved hypothetical protein	ND	(45)
SP1653	*vgcL*	0.949	0.538	ABC transporter, ATP-binding protein	ND	(46)
SP0746	*clpP*	0.822	0.226	ATP-dependent Clp protease	ND	(47)
SP2239	*htrK*	0.765	0.069	Serine protease	ND	(48)
SP0375	*gnd*	1.240	0.065	6-Phosphogluconate dehydrogenase	ND	(49)
SP0966	*pavA*	1.305	0.230	Adherence and virulence protein A	1.00	(50)
SP1128	*eno*	1.351	0.084	Enolase	1.58	(45)
SP0499	*pgK*	1.639	0.132	Phosphoglycerate kinase	1.54	(51)
SP2012	*gapA*	1.647	0.007	Glyceraldehyde 3-phosphate dehydrogenase	1.61	(52)
SP0314	*hyl*	1.725	0.042	Hyaluronidase	1.61	(53)
SP2185	*glpO*	2.850	0.031	Glycerol uptake facilitator protein	4.05	(54)
SP1004	*phtE*	3.049	0.005	Conserved hypothetical protein	1.58	(55)
SP1923	*ply*	3.541	0.005	Pneumolysin	3.45	(55)
SP1175	*phpB*	4.227	0.006	Conserved domain protein	1.67	(56)
SP1174	*phpA*	6.641	0.009	Conserved domain protein	1.44	(56)
SP1003	*phtD*	7.383	0.036	Conserved hypothetical protein	2.29	(55)
SP1687	*nanB*	8.947	0.005	Neuraminidase B	3.11	(57)

**Table 2:** Expression of choline-binding protein members and cell wall-anchoring protein genes in wild-type TIGR4 and TIGR4Δ*lgt* pneumococci.

	**Locus tag**	**Gene**	**Ratio (Δlgt/WT)**	***P*-value**	**Description**	**qPCR**
Choline-binding proteins	SP0390	*cbpG*	0.790	0.036	Choline-binding protein G	1.24
	SP0391	*cbpF*	0.764	0.017	Choline-binding protein F	1.01
	SP2201	*cbpD*	0.846	0.030	Choline-binding protein D	0.95
	SP1937	*lytA*	0.959	0.030	Autolysin	1.31
	SP0930	*cbpE*	0.922	0.033	Choline-binding protein E	1.41
	SP0667	*cbpL*	1.145	0.011	Choline binding protein L	1.01
	SP0965	*lytC*	1.253	0.124	Lysozyme	1.12
	SP0378	*cbpJ*	1.225	0.004	Choline-binding protein J	1.45
	SP2190	*cbpA*	1.220	0.006	Choline-binding protein A	1.53
	SP0377	*cbpC*	1.162	0.242	Choline-binding protein C	1.02
	SP1573	*lytB*	1.461	0.093	Endo-beta-*N*-acetylglucosaminidase	1.49
	SP0117	*pspA*	1.096	0.566	Pneumococcal surface protein A	1.22
	SP2136	*pcpA*	2.060	0.040	Choline-binding protein PcpA	1.38
	SP0069	*cbpI*	5.329	0.045	Choline-binding protein I	8.48
Cell wall-anchoring proteins	SP2145		1.415	0.018	Antigen, cell wall surface anchor family	ND
	SP0082		0.625	0.005	Cell wall surface anchor family	ND
	SP0368		1.795	0.002	Cell wall surface anchor family	ND
	SP0462		3.603	0.002	Cell wall surface anchor family	ND
	SP0463		3.530	0.003	Cell wall surface anchor family	ND
	SP0465		4.826	0.001	Cell wall surface anchor family	ND
	SP0667		1.145	0.011	Putative pneumococcal surface protein	ND
	SP1492		0.747	0.067	Cell wall surface anchor family	ND
	SP1772		1.261	0.136	Cell wall surface anchor family	ND
	SP1833		0.465	0.001	Cell wall surface anchor family	ND
	SP1992		0.797	0.017	Cell wall surface anchor family	ND

Lipoproteins are known to be potential inflammatory stimulants via the activation of TLR2. Various Gram-positive bacteria deficient in *lgt* have shown significant loss or complete abolition of inflammation-stimulating activity (29, 37). To determine the inflammation-stimulating activity of TIGR4Δ*lgt*, mouse RAW264.7 cells were infected with either TIGR4 or TIGR4Δ*lgt* for 12 h, and the production of nitrite and TNF-α was then examined (Figures 1B,C). TIGR4 treatment significantly upregulated levels of nitrite and TNF-α expression in a dose-dependent manner. By contrast, nitrite, and TNF-α production was not induced in cells infected with TIGR4Δ*lgt*, indicating that TIGR4Δ*lgt* failed to produce mature functional lipoproteins known to be responsible for stimulating inflammation. To examine whether the reduction in the inflammation-stimulating activity of TIGR4Δ*lgt* was due to the loss of TLR2-stimulating activity, mouse peritoneal macrophages isolated from wild-type (WT), TLR2-deficient (ΔTLR2), or TLR4-deficient (ΔTLR4) mice were treated with TIGR4 or TIGR4Δ*lgt*, and the level of TNF-α production was measured (Figure 1D). As expected, TIGR4 treatment induced TNF-α production in cells from WT and ΔTLR4 mice but not in cells from ΔTLR2 mice. However, TIGR4Δ*lgt* treatment produced no or significantly lower levels of TNF-α in cells from all three groups, suggesting that pneumococcus-induced expression of functional TLR2 agonists is dependent on Lgt and that TIGR4Δ*lgt* is likely defective in the production of TLR2 ligands.

Previous studies have suggested that LTA is another dominant TLR2 coactivator in Gram-positive bacteria (58, 59). Similar to *lgt* deletion, mutant bacteria lacking LTA elicit significantly reduced inflammatory activities in a variety of immune cells (60). Since pneumococci also express LTA, we questioned whether the lack of *lgt* in TIGR4 might affect the expression of LTA, thus affecting its ability to activate TLR2-mediated inflammation. Consequently, expression levels of LTA in the culture supernatants and cell walls of TIGR4 and TIGR4Δ*lgt* were measured. As shown in Figure 1E, the expression level of LTA in the culture supernatant of TIGR4Δ*lgt* was noticeably lower than that in the culture supernatant of TIGR4 based on Western blotting analysis using anti-phosphocholine antibody. LTA was further isolated from three different batches of pneumococcal cell wall via butanol extraction (Figure 1F). The average amount of LTA produced by TIGR4Δ*lgt* was about 53% less than that produced by its parent strain. These results indicate that deletion of *lgt* in pneumococci may impair host inflammatory responses owing to the synthesis of non-functional lipoproteins and the downregulation of LTA biosynthesis.

### Durable Colonization and Attenuated Pathogenicity of TIGR4Δ*lgt*

**Figure 2:**
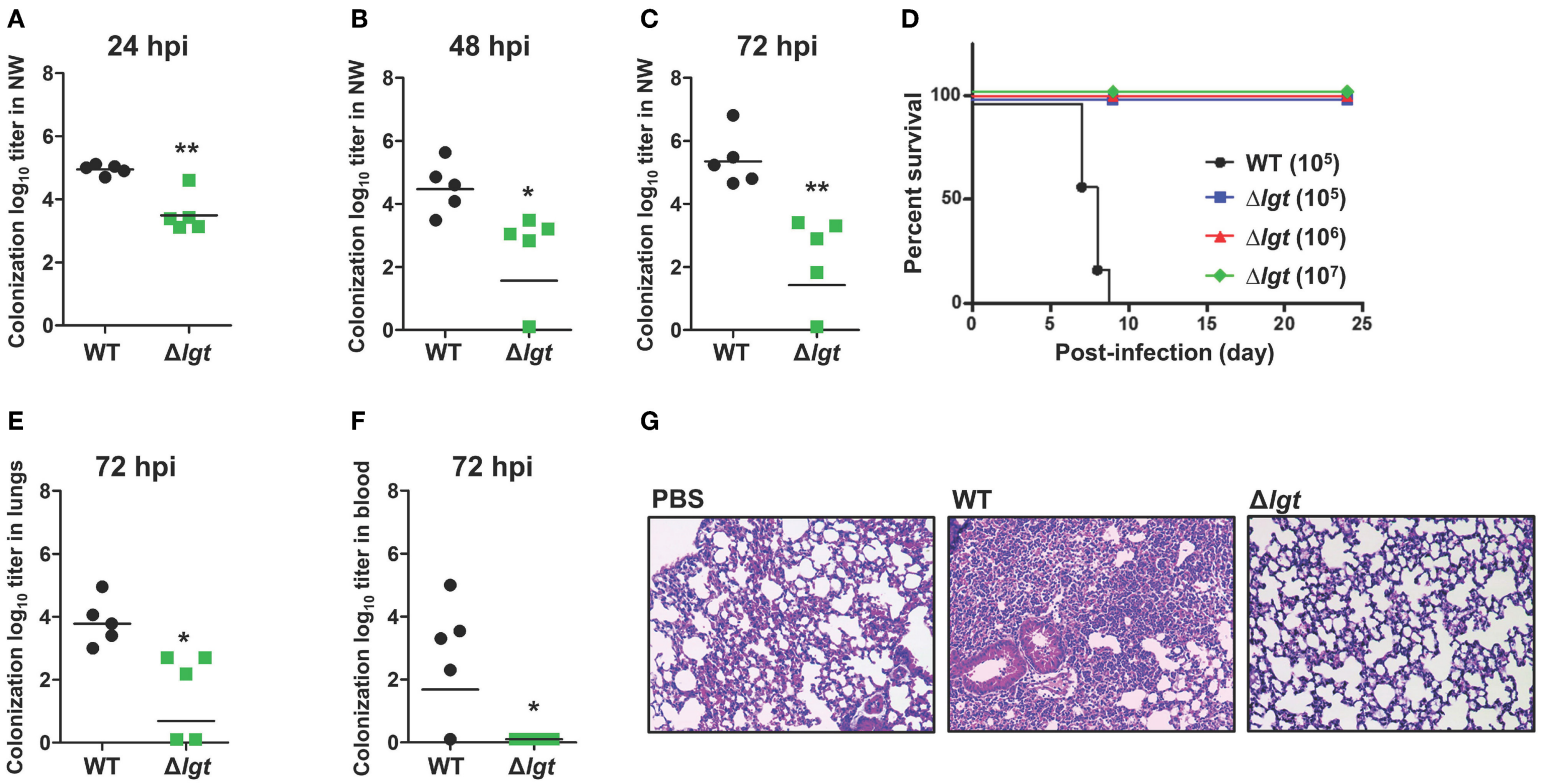
Durable Colonization of TIGR4Δ*lgt* in the Nasopharynx. **(A–C)** Pneumococcal colonization of the nasopharynx in mice (*n* = 5 per group) inoculated intranasally (i.n.) with either wild-type (WT) or Δ*lgt* TIGR4 (10^7^ CFU) at 24 h **(A)**, 48 h **(B)**, and 72 h **(C)** after inoculation. **(D)** Survival of mice (*n* = 10 per group) inoculated i.n. with either WT (10^5^ CFU) or Δ*lgt* TIGR4 (10^5^, 10^6^, or 10^7^ CFU). **(E,F)** Pneumococcal numbers in serially diluted lung tissue homogenates **(E)** and blood **(F)** from mice (*n* = 5 per group) inoculated i.n. with either WT or Δ*lgt* TIGR4 (10^7^ CFU) at 3 days after infection. **(G)** Hematoxylin & eosin (H&E) staining of lungs from mice inoculated with WT or Δ*lgt* TIGR4 (10^7^ CFU). ^*^P < 0.05 and ^**^P < 0.01 compared with the WT-infected mouse group.

To maximize immune responses, live attenuated vaccines should be able to effectively and durably colonize the mucosa without being invasive (19). To examine the role of *lgt* in colonization, mice were i.n. inoculated with either TIGR4 or TIGR4Δ*lgt* [10^7^ colony forming units (CFU)], and the number of bacteria colonizing the nasopharynx was measured in nasal washes at 24, 48, and 72 h post-infection (hpi) (Figures 2A–C). A significant reduction in the number of colonized bacteria was observed in TIGR4Δ*lgt-*inoculated mice compared to that in TIGR4-inoculated mice. However, TIGR4Δ*lgt* effectively colonized the nasopharynx for more than 3 days. No viable TIGR4Δ*lgt* was detected at 14 days post-infection (dpi) (data not shown).

To determine the invasiveness of TIGR4Δ*lgt*, mice were i.n. inoculated with TIGR4 (10^5^ CFU) or TIGR4Δ*lgt* (10^5^, 10^6^, or 10^7^ CFU), and mouse survival was recorded for 24 days. As shown in Figure 2D, all mice infected with TIGR4Δ*lgt* survived for more than 24 days, even at infection levels of 10^7^ CFU. However, all mice inoculated with 10^5^ CFU of TIGR4 died by 9 dpi. No significant weight loss or noticeable disease symptoms were observed in mice inoculated with TIGR4Δ*lgt*, suggesting a lack of systemic toxicity (data not shown). Next, the dissemination of bacteria into the lungs and blood was measured at 3 dpi with TIGR4 or TIGR4Δ*lgt* (10^7^ CFU). The bacterial load in the lungs was significantly reduced in TIGR4Δ*lgt*-inoculated mice compared to that in TIGR4-inoculated mice (Figure 2E). No dissemination of TIGR4Δ*lgt* into the bloodstream was observed for up to 14 days (Figure 2F and data not shown). Consistent with findings from survival and microbiological analysis, lungs from mice infected with TIGR4 showed marked pathological changes in inflammation, including enhanced neutrophil infiltration, loss of alveolar architecture, and red blood cell leakage (Figure 2G). However, although a modest number of TIGR4Δ*lgt* was disseminated into the lungs, no significant pathological change was found in the lungs of TIGR4Δ*lgt*-infected mice (Figure 2G). These data suggest that TIGR4 was successfully attenuated by deleting *lgt* but that TIGR4Δ*lgt* can still colonize the nasopharynx.

### Protective Humoral Immune Responses Induced by TIGR4Δ*lgt* Vaccination

**Figure 3:**
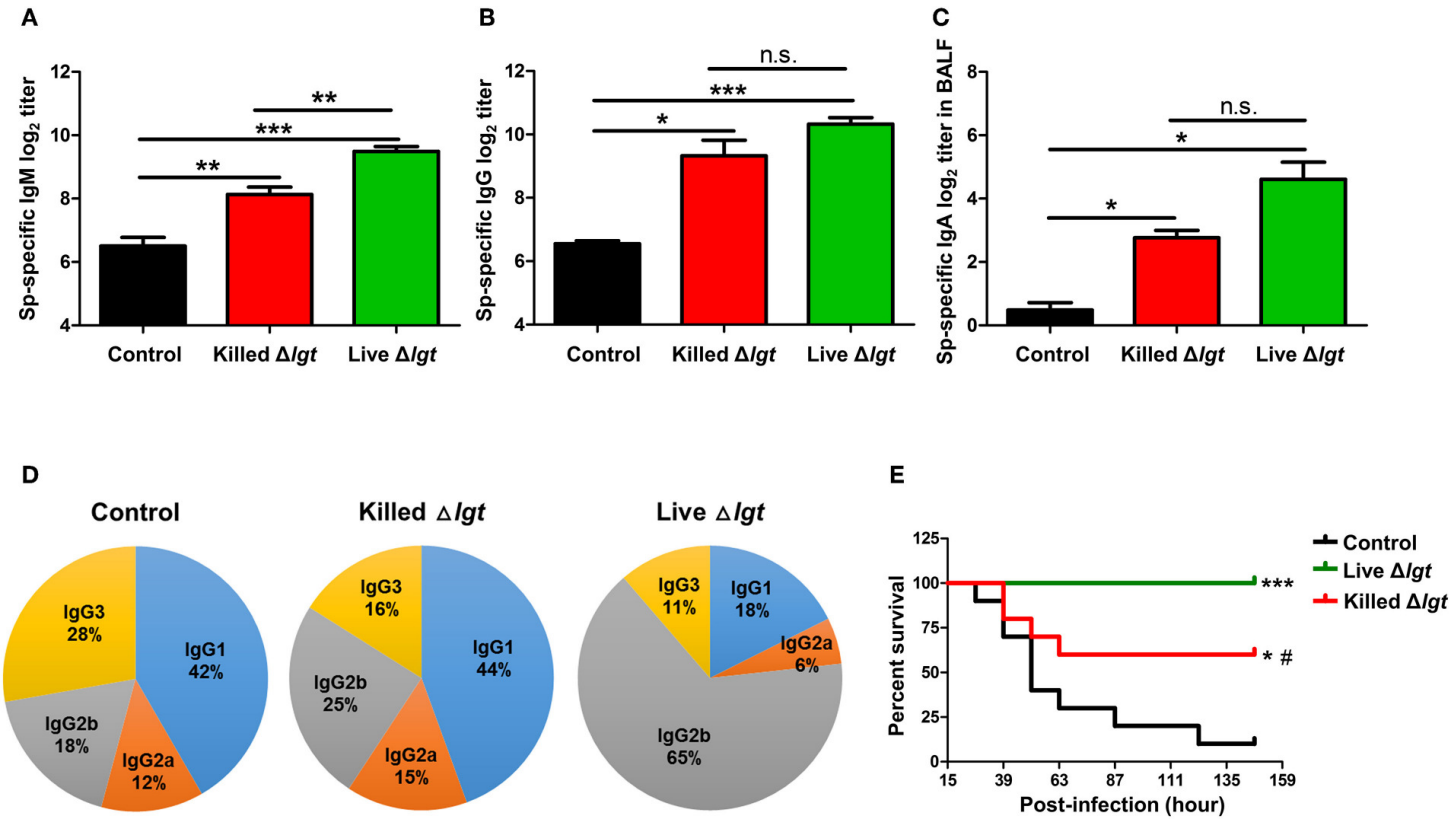
Humoral and Mucosal Protective Immune Responses Induced by Live Δ*lgt* Vaccination. **(A–D)** Levels of *Sp*-specific serum IgM **(A)**, serum IgG **(B)**, and BALF IgA **(C)** and ratios of serum IgG subclasses **(D)** determined 10 days after second immunization in mice (*n* = 5 per group) i.n. immunized twice with killed Δ*lgt* or live Δ*lgt* at 14-day intervals. Data in **(A–C)** indicate mean values ± S.D. n.s.; not significant, ^*^P < 0.05, ^**^P < 0.01, and ^***^P < 0.001 compared to PBS-inoculated controls. **(E)** Survival of mice (*n* = 5 per group) i.n. immunized twice with killed Δ*lgt* or live Δ*lgt* at 14-day intervals, followed by intraperitoneal (i.p.) challenge with 10^7^ CFU of wild-type TIGR4. ^*^P < 0.05 and ^***^P < 0.001 compared to control groups immunized with PBS. ^#^*P* < 0.05 compared to the live Δ*lgt*-vaccinated group.

We next evaluated the immunogenicity of live TIGR4Δ*lgt* (live Δ*lgt*) and compared it with that of inactivated TIGR4Δ*lgt* (killed Δ*lgt*). Mice were i.n. immunized with either live or killed Δ*lgt* twice at 14-day intervals (days 0 and 14). Pneumococcal-specific immunoglobulin levels in the serum and bronchoalveolar lavage fluid (BALF) were then measured at 10 days after the second immunization. As shown in Figures 3A,B, both live and killed Δ*lgt* vaccines induced significantly higher serum levels of pneumococcal-specific IgM (*Sp*-specific IgM) and *Sp*-specific IgG than inoculation with PBS (control group). Moreover, *Sp*-specific IgM levels in the sera of live Δ*lgt-*immunized mice were significantly higher than those in the sera of mice immunized with killed Δ*lgt*. However, no differences in *Sp*-specific IgG levels were found between the live and killed Δ*lgt* immunization groups. In addition, significant induction of *Sp*-specific IgA was found in BALF from both the live and killed Δ*lgt-*immunized groups (Figure 3C), indicating that both live and killed Δ*lgt* vaccines effectively induced mucosal immune responses.

Distinct IgG subclasses have different efficacies in terms of their functions, such as serum bactericidal activity and opsonophagocytosis. Therefore, IgG subclass distributions may contribute to the protective effects of vaccination. Despite the fact that similar levels of serum IgG were elicited by live and killed Δ*lgt*, IgG subclass analysis showed markedly different IgG subclass patterns between the two immunization groups (Figure 3D). Specifically, IgG1 (44%) was predominant, followed by IgG2b (25%), IgG3 (16%), and IgG2a (15%), in the sera of mice immunized with killed Δ*lgt*, whereas a strong shift to an IgG2b (65%) antibody response was observed in mice immunized with live Δ*lgt*.

Next, we determined the immunological efficacy of the live vs. killed Δ*lgt* vaccines by measuring their protective effects against detrimental challenge with TIGR4. Mice immunized with either live or killed Δ*lgt* were i.n. challenged with WT TIGR4 (10^7^ CFU), and mouse survival was recorded 10 days after the second immunization. As shown in Figure 3E, 90% of control mice (PBS-immunized) died by 5 dpi, whereas 60% of mice immunized with killed Δ*lgt* and 100% of mice immunized with live Δ*lgt* survived for more than 6 dpi. Taken together, these findings demonstrate that live Δ*lgt* vaccination induces high levels of IgM and IgG2-prone humoral immune responses, providing protection against detrimental challenge with its parent strain, TIGR4, in mice.

### Serotype-Independent Protection Conferred by TIGR4Δ*lgt* Vaccination

**Figure 4:**
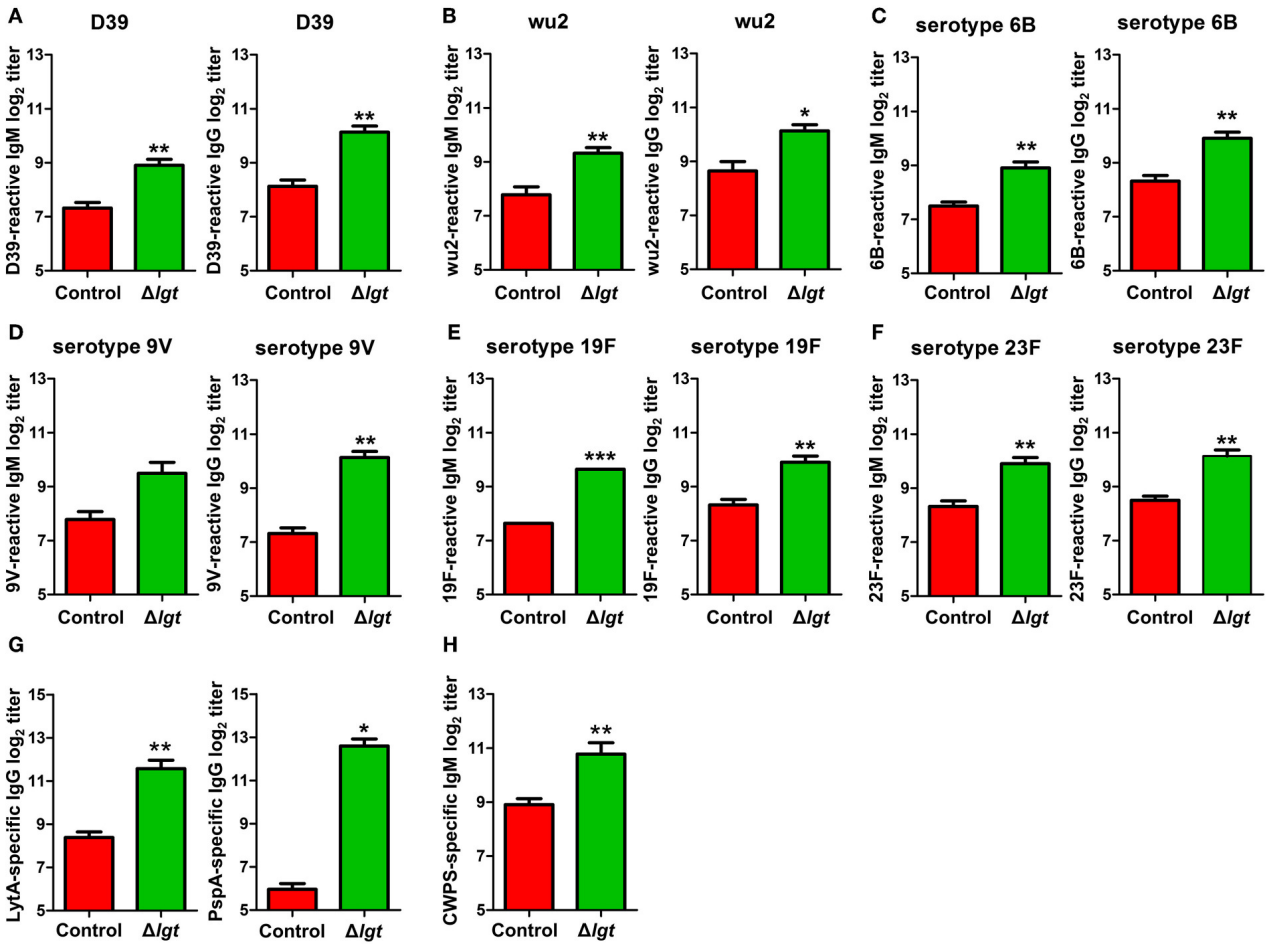
Cross-reactive antibody production following immunization with TIGR4Δ*lgt*. Serum levels of IgM and IgG reactive to serotypes 2 (D39; **A**), 3 (wu2; **B**), 6B **(C)**, 9V **(D)**, 19F **(E)**, and 23F **(F)** and serum levels of LytA-, PspA-, and cell wall PS-specific IgG **(G,H)** were determined at 10 days after the second immunization in mice (*n* = 5 per group) i.n. immunized twice with Δ*lgt* (10^6^ CFU) at 14-day intervals. ^*^P < 0.05, ^**^P < 0.01 and ^***^P < 0.001 compared with control groups immunized with PBS.

To overcome the major limitations of currently available pneumococcal vaccines, vaccines should ideally be designed to provide serotype-independent, broad-spectrum protection against heterologous pneumococcal serotypes. Therefore, we assessed the cross-reactivity of the antibodies induced by TIGR4Δ*lgt* to different pneumococcal serotypes. As shown in Figures 4A–F, the IgM and IgG antibodies induced by TIGR4Δ*lgt* showed capsular serotype-independent reactivities to heterologous pneumococcal serotypes (STs), including ST2 (D39), ST3 (wu2), ST6B, ST9V, ST19F, and ST23F. In addition, we investigated production of the antibodies against conserved pneumococcal surface antigens such as LytA, an autolysin, and pneumococcal surface protein PspA, a component of transport system. TIGR4Δ*lgt* immunization produced antibodies against LytA and PspA as well as antibodies against purified cell wall PS (Figures 4G,H).

**Figure 5:**
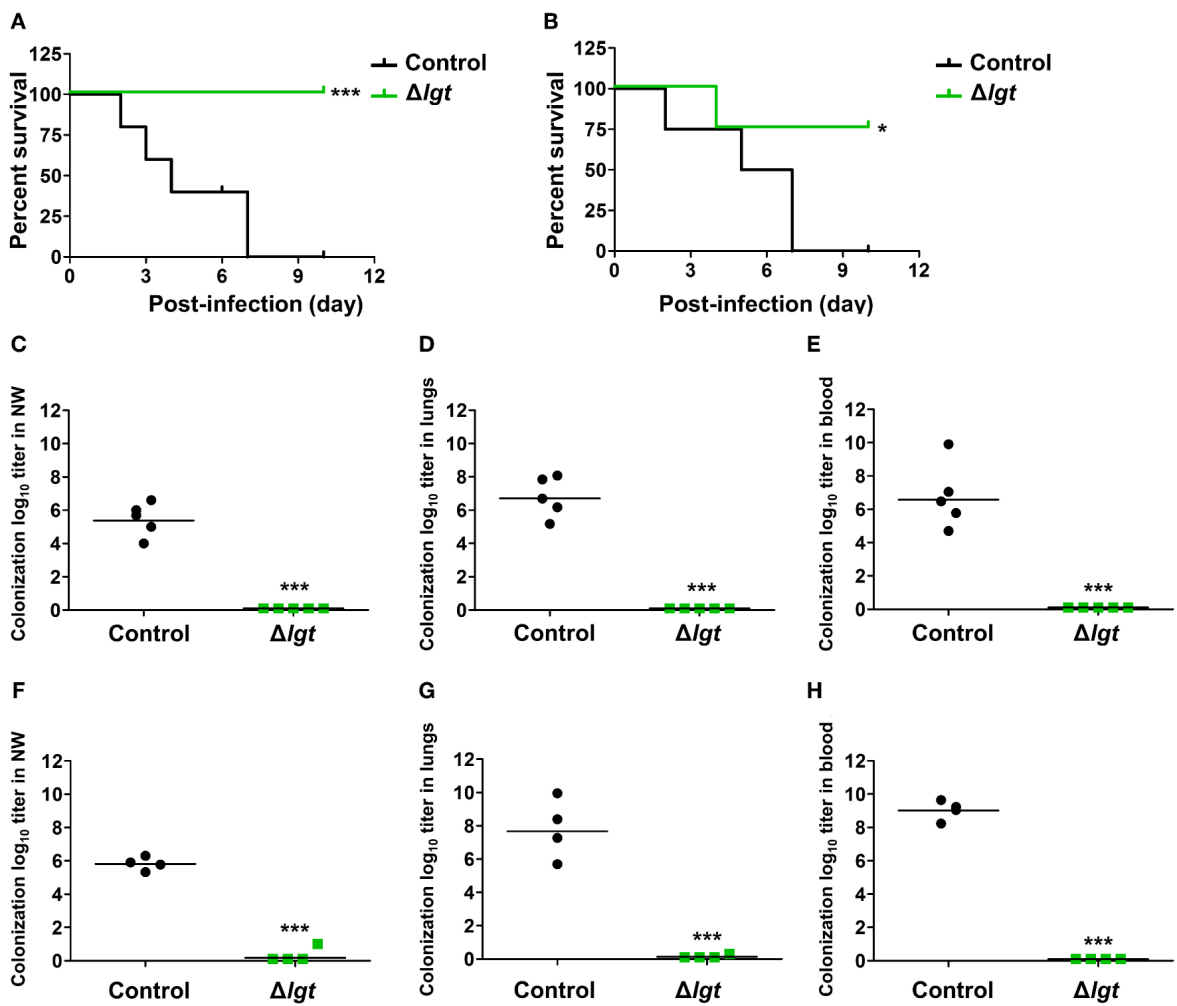
Serotype-independent protection conferred by TIGR4Δ*lgt* vaccination. **(A,B)** Survival of mice (*n* = 5) i.n. immunized with Δ*lgt* (10^6^ CFU) twice at 14-day intervals, followed by i.n. challenge with 10^8^ CFU of wu2 **(A)** or D39 **(B)** strains. **(C–H)** Pneumococcal numbers counted within serially diluted nasal washes **(C,F)**, lung tissue homogenates **(D,G)**, and blood **(E,H)** from Δ*lgt* (10^6^ CFU)-immunized mice challenged with wu2 **(C–E)** or D39 **(F–H)**. ^*^P < 0.05 and ^***^P < 0.001 compared to the control group immunized with PBS.

Next, we investigated whether TIGR4Δ*lgt* vaccination provided cross-protection against different serotypes. Mice were i.n. immunized with TIGR4Δ*lgt* twice at 14-day intervals (day 0 and day 14) and challenged with a lethal dose of wu2 (Figure 5A) or D39 (Figure 5B) at 10 days after the second immunization. All mice challenged with wu2 without immunization (control) died by 7 dpi (0% survival). However, immunization with TIGR4Δ*lgt* resulted in complete protection against wu2 challenge (100% survival) (Figure 5A). Similarly, D39 challenge resulted in 100% mortality for the unimmunized group by 7 dpi, while 75% of mice immunized with TIGR4Δ*lgt* survived for more than 10 dpi, indicating serotype-independent protection offered by TIGR4Δ*lgt* vaccination (Figure 5B). Bacterial loads in the nasopharynx, lungs, and blood of mice were also significantly reduced by TIGR4Δ*lgt* vaccination following detrimental challenge with either wu2 (Figures 5C–E) or D39 (Figures 5F–H).

## Discussion

After adding PCVs to the immunization schedule for the pediatric population, the incidence of invasive pneumococcal diseases in both vaccinated children and unvaccinated individuals of all ages has been significantly reduced (9, 10). However, non-vaccine serotypes and new serotypes have emerged as major causes of pneumococcal diseases (11, 12, 61). To overcome these limitations, the development of innovative vaccines including protein-based vaccines, inactivated whole-cell-based vaccines, and live attenuated vaccines capable of covering all or most pneumococcal serotypes is actively being pursued (16, 19, 20, 62). Among these vaccines, live attenuated vaccines offer several advantages as cross-protective, broad-spectrum pneumococcal vaccines, such as the effective induction of both memory and cell-mediated immune responses against common and important pneumococcal antigens (21). Despite these benefits, a degree of unpredictability raises safety and stability concerns regarding the widespread adaptation of live attenuated vaccines (63). For example, Bacillus Calmette-Guerin (BCG) vaccination for tuberculosis can result in severe local inflammation or disseminated infection (64). In the current study, we developed a live attenuated vaccine for pneumococcal infections by deleting the *lgt* gene in the encapsulated pneumococcal strain TIGR4 (TIGR4Δ*lgt*), resulting in significant attenuation of invasiveness and inflammation-activating abilities. However, TIGR4Δ*lgt* was able to colonize the mouse nasopharynx long enough to elicit mucosal antibody responses and IgG2b-dominant, Th1-biased systemic immune responses that were cross-reactive to heterologous pneumococcal serotypes. Moreover, the live TIGR4Δ*lgt* vaccine provided better protection than the killed whole-cell-based vaccine against subsequent detrimental challenge with the parental WT (TIGR4) strain.

Lipoproteins comprise the largest group of bacterial surface proteins and contribute significantly to bacterial adaptation to environmental changes, uptake of nutrients, and adherence to host membranes during infection (24). The importance of bacterial lipoproteins in TLR2-mediated immune recognition and pro-inflammatory responses has been extensively investigated using *lgt-*deficient pathogens (27, 65, 66). Both the present and previous studies showed that *lgt-*deficient pneumococci cannot activate TLR2-dependent cellular responses in macrophages. Although TLR2 responses are required for stimulating the innate and adaptive immune responses (e.g., Th1, Th2, Th17, Treg) of the host (67–69), our data showed that live Δ*lgt* vaccination induces IgG2-dominant humoral responses, which are prone to Th1-biased responses in mice. Unlike in human, in which IgG1 is more effective in opsonization and FcγR-mediated pneumococcal phagocytosis, IgG2 functions as a highly effective opsonin and more significantly involved in opsonophagocytosis and serum bactericidal activities than IgG1 in mouse (70, 71). IgG2, both IgG2a and 2b, is a surrogate marker for Th1 response and highly activate complements and opsonophagocytosis than IgG1 (70, 72). Such a significant change in IgG subclass pattern may contribute to the enhanced protection induced by live Δ*lgt* vaccination. In addition, a significantly higher level of IgM, which is a major Ig class responsible for complement activation, and IgM-mediated complement activation may also contribute to the enhanced protective effects observed in live Δ*lgt* vaccination. Previous studies have also indicated that staphylococcal and pneumococcal Δ*lgt* strains elicit significant upregulation of proinflammatory cytokines (IL-8, IL6, and TNF-α) in human leukocytes, though it is lower than that of WT, which may be mediated by pathogen-associated molecular patterns (PAMPs) other than lipoproteins, including peptidoglycan, pneumolysin, and LTA (40, 58, 73). Consequently, we hypothesized that live TIGR4Δ*lgt* may still have the ability to stimulate adaptive immune responses via lipoprotein-TLR2-independent mechanisms. In addition, IFN-γ production is known to regulate pneumococcal infections and T cell-mediated immunological memory responses (74, 75). Similar to the results of a previous study (37), our observations showed that live TIGR4Δ*lgt* vaccination elicited significantly higher levels of IFN-γ production by splenocytes than killed TIGR4Δ*lgt* vaccination (data not shown), suggesting that the superior protection against pneumococcus conferred by live TIGR4Δ*lgt* vaccination may be due to the IgG2b/IgG2a-predominant Th1 response via the IFN-γ pathway. However, further insight into how lipoprotein-deficient pneumococci modulate Th1-mediated host defenses is warranted.

Pneumococcal colonization leads to naturally acquired mucosal events that induce both Th17 cell-mediated and humoral adaptive immune responses (76, 77). Thus, prolonged colonization of the nasopharynx may be another critical factor in the development of effective pneumococcal live attenuated vaccines. Many attenuated pneumococcal strains lacking major virulence genes, such as *lytA, pspA*, and *pspC*, have failed to establish successful nasopharyngeal colonization (78, 79). However, while the TIGR4Δ*lgt* developed in this study showed significantly reduced colonization of the mouse nasopharynx compared to that of its parent strain, it still colonized long enough to induce mucosal and systemic humoral antibody responses against various lethal pneumococcal strains. Despite such prolonged colonization, TIGR4Δ*lgt* did not result in significant inflammation or dissemination into the lungs or bloodstream, even at a multiplicity of infection >1,000 times the median lethal dose of the parental strain. Such localized colonization indicates the highly successful attenuation of invasiveness, thus ensuring the great potential of TIGR4Δ*lgt* as a vaccine candidate.

Since airway is a major route of pneumococcal infection, alveolar macrophages play a role on defense against pneumococcus. Although the level of IgA, which is a major Ig class in the airway, was not significantly different in BALF between killed and live TIGR4Δ*lgt* vaccination, live TIGR4Δ*lgt* immunization more effectively removed pneumococcus from the alveolar airway. These findings may suggest that alveolar macrophage-mediated phagocytosis may play roles on enhanced protection observed in live TIGR4Δ*lgt* immunization. Although TIGR4Δ*lgt* failed to produce mature lipoproteins functioning as adhesins, our microarray data indicated that most cell surface-anchoring proteins (e.g., choline-binding proteins) were expressed at levels similar to that in the parental TIGR4 strain, likely facilitating sufficient colonization. Thus, prolonged colonization of TIGR4Δ*lgt* may elicit cross-reactive, pneumococcal-specific IgG and IgM production and, therefore, more efficient Th1-biased antibody responses, providing improved protective immunity against multiple serotypes.

In conclusion, this study demonstrated that the deletion of a single pneumococcal “master” gene, *lgt*, results in the creation of a live attenuated vaccine with attenuated virulence and invasive ability. Furthermore, this study showed that TIGR4Δ*lgt* has the potential to induce broad-spectrum, serotype-independent, protective immunity against life-threatening pneumococcal infections. Since conventional serotype-based vaccines have limitations, including a lack of protection against non-vaccine serotypes, the need for multiple doses and adjuvants, and high cost, the findings of this study may be useful in the development of inexpensive, but highly efficacious, vaccines against pneumococcal infections. In a broader sense, the modulation of major metabolic regulators represents an emerging strategy in the design of immunologic agents.

## Ethics Statement

All animal experiments conducted in this study were approved by the Committee on the Use and Care of Animals at the Korea Atomic Energy Research Institute (KAERI) and performed according to accepted veterinary standards.

## Author Contributions

JL and HS conceived the study. A-YJ, KA, and HS designed the experiments. A-YJ, KA, YZ, HJ-J, JZ, and HS performed the experiments. A-YJ, KA, and HS analyzed and/or interpreted the data and contributed to discussion of the results, followed by writing and reviewing the manuscript. SH, HG, SL, JS, JL, and HS provided critical comments and contributed to discussion of the results, followed by writing and reviewing the manuscript.

### Conflict of Interest Statement

The authors declare that the research was conducted in the absence of any commercial or financial relationships that could be construed as a potential conflict of interest.
