# Supplementary material for: Serotype-Independent Protection Against Invasive Pneumococcal Infections Conferred by Live Vaccine With lgt Deletion
Source: Front Immunol. 2019 May 29;10:1212. doi: 10.3389/fimmu.2019.01212 (PMC6549034; doi:10.3389/fimmu.2019.01212)
Supplement: Supplementary file 1 [file Table_1.DOCX]

**Supplement Table 1. Strains and plasmids used in this study.**

| **Strain or plasmid** | **Relevant genotype** | **Source or reference** |
| --- | --- | --- |
| ***Escherichia coli*** |  |  |
| **DH5α** | F^-^r^-^m^+^Ø80d*lacZ*∆M15 | Gibco BRL |
| ***Streptococcus pneumoniae*** | |  |
| **TIGR4** | serotype 4 | ATCC |
| **TIGR4Δ*lgt*** | *lgt* deficient strain | current study |
| **D39** | serotype 2 |  |
| **Wu2** | serotype 3 |  |
| **MNZ1921** | serotype 6B clinical isolates |  |
| **MNZ1954** | serotype 9V clinical isolates |  |
| **MNZ3146** | serotype 19F clinical isolates |  |
| **MNZ2155** | serotype 23F clinical isolates |  |
| **Plasmid** |  |  |
| **pK326** | cloning vector | (69) |
| **pKO-lgt** | *lgt* suicide vector | Current study |
| **pET28_FLAG_** | expression vector with 3xFLAG-tag | (65) |
| **pET28_FLAG_:LytA** | vector for expression of 3xFLAG-tagged LytA_31-318_ | Current study |
| **pET28_FLAG_:PsaA** | vector for expression of 3xFLAG-tagged PsaA_22-309_ | Current study |
| **pET28_FLAG_:PspA** | vector for expression of 3xFLAG-tagged PspA_32-327_ | Current study |
