# Supplementary material for: Serotype-Independent Protection Against Invasive Pneumococcal Infections Conferred by Live Vaccine With lgt Deletion
Source: Front Immunol. 2019 May 29;10:1212. doi: 10.3389/fimmu.2019.01212 (PMC6549034; doi:10.3389/fimmu.2019.01212)
Supplement: Supplementary file 2 [file Table_2.DOCX]

**Supplement Table 2. Primers used in this study.**

| **Gene** | **Primer name** | | **Primer sequence (5’-3’)** |
| --- | --- | --- | --- |
| ***16S*** | 3028 | | AAG CAA CGC GAA GAA CCT T |
|  | 5028 | | GTC TCG CTA GAG TGC CAA C |
| ***NanB*** | 3029 | | CGT GGA GTG AGC CAA TTT TT |
|  | 5029 | | CGC AGG CAT AAC ATC AGC TA |
| ***PhpA*** | 3030 | | CGA GAA GAG ACC GCA AAG |
|  | 5030 | | TGG ATC CTG GAT TTT TCC AA |
| ***PhtD*** | 3031 | | CCA AGC ACT GAT ACG GAA GAG |
|  | 5031 | | AGG AGC CGG TTG ACT TTC TT |
| ***PhpB*** | 3032 | | AAC CGA CTC CGG AAC CTA GT |
|  | 5032 | | TCC CCA ACT TTT CGT ACC AG |
| ***Ply*** | 3033 | | CAG TCG CCT CTA TCC TGG AG |
|  | 5033 | | AGC CAA CAA ATC GTT TAC CG |
| ***PhtE*** | 3034 | | TAG TTT TCC GCC TGA ATT GG |
|  | 5034 | | CCC TGC ATG GAA AGG ATA GA |
| ***GlpO*** | 3035 | | TCC ATC TGC AGT TTC TCG TG |
|  | 5035 | | GCT CCT TCA GCC ATC TTA CG |
| ***PgK*** | 3036 | | CAG TCG TAT GGA ACG GAC CT |
|  | 5036 | | GCT GCA AGT CCT GGA AGA AC |
| ***Gap*** | 3037 | | CGC ATC AAC GAC CTT ACA GA |
|  | 5037 | | TTC TGG ATC ACG TTC AGC AG |
| ***Hyl*** | 3038 | | AGC GGA AGA AAC GAC TAC GA |
|  | 5038 | | GAG CTT TGC TTC CTG TCC AC |
| ***Eno*** | 3039 | | CTC GTG CTG CTG CTG ACT AC |
|  | 5039 | | GGA GCG TCA GAG TGA GAA CC |
| ***Cbpl*** | 3040 | | TGT GAA GAC AGG TTG GGT GA |
|  | 5040 | | ATA GCT CCT GAG CCG TCA AA |
| ***PcpA*** | 3041 | | CAT TGC CGT CCT TCT TCC TA |
|  | 5041 | | CCC AAC CAA CTA CTC CCT GA |
| ***PspA*** | 3042 | | CTC CAG CTC CAA AAC CAG AG |
|  | 5042 | | CGC CGT TAG CGT TTA GGT AG |
| ***LytC*** | 3043 | | GGC TAT ATG GCT CGG AAT GA |
|  | 5043 | | CCA GCC GAC ATT CAA ATC TT |
| ***CbpC*** | 3044 | | GGC CGA TTT GTT GAT AAG GA |
|  | 5044 | | CCG ACA ACC ATC TCT CCA AT |
| ***CbpA*** | 3045 | | AAG CAG AAA AAC CAG CTC CA |
|  | 5045 | | ATT GTT TTG GAG CCA TCC TG |
| ***CbpJ*** | 3046 | | AAA AAG GGG GAC CTC AAA GA |
|  | 5046 | | CCC CGT ACT TTC TTC CAA CA |
| ***LytB*** | 3047 | | AGC TAG TCC AGA GGG TGC AA |
|  | 5047 | | GTC GTC ACC TTG CTT TAG CC |
| ***PavA*** | 3048 | | GAA ATC CTG CAA ACC CAA GA |
|  | 5048 | | AAG GCT CTC CCA CCT GAT TT |
| ***CbpL*** | 3049 | | CGG ATA TTC ACG AGG GAG AA |
|  | 5049 | | AGC CGT TGA CTG TTT TGC TT |
| ***CbpE*** | 3050 | | ACG CAG CCA GCA AAG ACT AT |
|  | 5050 | | ATT TCA TTC CAA CCG ACA GC |
| ***LytA*** | 3051 | | CTA TGC AGC GGT TGA ACT GA |
|  | 5051 | | GCT AAT GCC CCA TTT AGC AA |
| ***CbpD*** | 3052 | | AAA ATG GGA GCC AGG AGA TT |
|  | 5052 | | CCG GAA TTT CAA AAC CAT TG |
| ***CbpF*** | 3053 | | AGG ATG GCA GTT TGT CCA AG |
|  | 5053 | | CCC TTG TGT GGA GCA GGT AT |
| ***CbpG*** | 3054 | | GGA GTA GCC GAT GGA ATG AA |
|  | 5054 | | CTT GGA CTA ACA GCC GGA AG |
| **Construction of TIGR4Δ*lgt*** | | | |
| **Lgt-F** | | AAG TTT TTT TTC CGG GTT CTT TTA AAA ATC AGT CAC AAG TAA GGT AGG GTT CAT ATG AAT ATC CTC CTT A | |
| **Lgt-R** | | GCA GTT CCT GTT TGT AGA TTT CAA TCT CTT TGC GCA GCG CGC GAA CTT CTT GTA GGC TGG AGC TGC TTC | |
